# Effects of *Nigella sativa* and *Lepidium sativum* on Cyclosporine Pharmacokinetics

**DOI:** 10.1155/2013/953520

**Published:** 2013-07-16

**Authors:** F. I. Al-Jenoobi, S. A. Al-Suwayeh, Iqbal Muzaffar, Mohd Aftab Alam, Khalid M. Al-Kharfy, Hesham M. Korashy, Abdullah M. Al-Mohizea, Abdul Ahad, Mohd Raish

**Affiliations:** ^1^Department of Pharmaceutics, College of Pharmacy, King Saud University Riyadh, P.O. Box 2457, Riyadh 11451, Saudi Arabia; ^2^Department of Pharmaceutical Chemistry, College of Pharmacy, King Saud University Riyadh, Riyadh 11451, Saudi Arabia; ^3^Department of Clinical Pharmacy, College of Pharmacy, King Saud University Riyadh, Riyadh 11451, Saudi Arabia; ^4^Department of Pharmacology, College of Pharmacy, King Saud University Riyadh, Riyadh 11451, Saudi Arabia

## Abstract

The present study was conducted to investigate the effects of *Nigella sativa* and *Lepidium sativum* on the pharmacokinetics of cyclosporine in rabbits. Two groups of animals were treated separately with *Nigella sativa* (200 mg/kg p.o.) or *Lepidium sativum* (150 mg/kg p.o.) for eight consecutive days. On the 8th day, cyclosporine (30 mg/kg p.o.) was administered to each group one hour after herbal treatment. Blood samples were withdrawn at different time intervals (0.0, 0.5, 1.0, 1.5, 2.0, 3.0, 4.0, 6.0, 8.0, 12, and 24 hrs) from marginal ear vein. Cyclosporine was analyzed using UPLC/MS method. The coadministration of *Nigella sativa* significantly decreased the *C*_max_ and AUC_0−*∞*_ of cyclosporine; the change was observed by 35.5% and 55.9%, respectively (*P* ≤ 0.05). *Lepidium sativum* did not produce any significant change in *C*_max_ of cyclosporine, although its absorption was significantly delayed compared with control group. A remarkable change was observed in *T*_max_ and AUC_0−*t*_ of *Lepidium sativum* treated group. Our findings suggest that concurrent consumption of *Nigella sativa* and *Lepidium sativum* could alter the pharmacokinetics of cyclosporine at various levels.

## 1. Introduction

Based on the belief that natural medicines are much safer than synthetic medicines, the use of complementary and alternative medical therapies has become a common trend in developing countries [1]. According to World Health Organization (WHO), a significant percentage of population living in developing countries relies on traditional medicine for their primary health care needs [2]. The literature based on *in vitro* and *in vivo* investigations suggests that concomitant administration of natural products and prescription drugs may modulate drug pharmacokinetics, which may lead to toxic or subtherapeutic incidences. The incidences of herb-drug interactions have grown serious concern especially when the drug has a narrow therapeutic index, for example, warfarin, cyclosporine, and digoxin [3, 4]. 

Herbal remedies may contain multiple active constituents such as alkaloids, flavonoids, glycosides, anthraquinones, saponins, and others, which have the potential to interact with therapeutic drugs. It has been demonstrated that many herbal products elicit different effects on CYP-mediated pathways [5–8]. There is numerous evidence of drug interactions involving cytochrome-P450 (CYP) drug metabolizing enzymes and p-glycoprotein (P-gp) efflux transporters [9]. Therefore, consumption of herbs that are capable of modulating CYP or drug transporters may cause clinically relevant herb-drug interactions by altering the bioavailability and pharmacokinetics of drugs [10]. 

Cyclosporine A (CsA) is a cyclic polypeptide and widely used as an immunosuppressant in organ transplantation [11, 12]. It has been reported as a narrow therapeutic index drug that may cause severe nephritis and neurotoxicity at high concentration and fails to produce pharmacological effect below minimum effective concentration [13]. CsA is a substrate of CYP3A and P-gp; therefore, it is expected that concomitant administration of cyclosporine with CYP3A/P-gp modulators can alter the pharmacokinetics of cyclosporine and may produce clinically significant interactions [12, 14]. The pharmacokinetic interactions of CsA with many herbs have been reported. Some of these herbs are Rhei Rhizoma extract, Quercetin, St John's wort, *Schisandra sphenanthera* extract, Ginger rhizome, Ginkgo and Onion [14–18]. 

*Nigella sativa* (Ranunculaceae family) also known as black seed or “blessed seed,” is an annual herb that grows in countries bordering the Mediterranean Sea, Pakistan, and India [19]. Black seed has been used as a natural remedy over thousand years to promote health and treat diseases. The oil of black seed had been used as an Arab traditional medicine for the treatment of arthritis, lung diseases, and hypercholesterolemia [20]. Recent studies also showed that the oil of *Nigella sativa* may be considered as a potential adjuvant for the therapy of rheumatoid arthritis [21]. *Lepidium sativum* Linn., commonly known as “garden cress,” belongs to Cruciferae family. It has been used for respiratory disorders, vitamin C deficiency, constipation, poor immunity, and as a diuretic. The practitioners of Indian medicine consider its seeds useful in dysenteric diarrhea as well as in febrile and catarrhal infections. The antidiarrheal and antispasmodic activities of garden cress are species dependent. Its antispasmodic effect is probably through combination of multiple pathways including activation of K^(+)^ channels and inhibition of muscarinic receptors, Ca^(++)^ channels, and PDE enzyme [22, 23]. *Lepidium sativum* seeds are also considered to be lactagogue and administered after boiling with milk [24, 25].

## 2. Materials and Methods

### 2.1. Chemicals and Reagents

Working standard of cyclosporine A was obtained as USP reference standard (Rockville, MD, USA), and internal standard cyclosporine D was purchased from Santa Cruz Biotechnology Inc. Neoral oral solution containing 100 mg/mL cyclosporine A (Novartis Pharma, Switzerland, MFD: 12-2010; EXP: 11-2013; LOT: H5108A) was obtained from pharmacy of King Khalid University Hospital, Riyadh, Saudi Arabia. The seeds of *Nigella sativa* (black seed) and *Lepidium sativum* (garden cress) were purchased in dry form from a local market and authenticated by a taxonomist at the College of Pharmacy, King Saud University, Riyadh, Saudi Arabia. Ammonium acetate and zinc sulphate were purchased from Fluka Chemika (Switzerland) and Merck (Germany), respectively. All aqueous solutions were prepared using purified water filtered by Milli-QR Gradient A10R (Millipore, Molsheim Cedex, France), having pore size 0.22 *μ*m. All other materials were of analytical grade when appropriate.

### 2.2. Animals and Study Protocol

Ten healthy rabbits of either sex weighing between 3.0 and 4.0 kg were obtained from College of Pharmacy, Animal Care and Use Center, King Saud University, Riyadh, Saudi Arabia. The animals were randomly divided into two groups (5 each). Animals were maintained in accordance with the recommendations of the “Guide for the Care and Use of Laboratory Animals” approved by the center. All rabbits were maintained under standard laboratory conditions of a 12-hour light/dark cycle at 25°C ± 2°C. The animals were given pellet diet with water *ad libitum *and fasted overnight prior to the experiments. 

In a crossover design, overnight fasted rabbits (*n* = 5) were placed in individual restrainer. A polyethylene catheter (0.56 mm i.d., 0.98 mm o.d.) was inserted into the ear vain of each rabbit to collect blood samples. CsA (30 mg/kg, p.o.) was given orally to each rabbit and blood samples (~2 mL) were collected into heparinized vacutainer tubes at 0.0, 0.5, 1.0, 1.5, 2.0, 3.0, 4.0, 6.0, 8.0, 12, and 24 hour intervals. Blood samples were stored at −80°C until analyzed. Thereafter, rabbits were treated with saline suspension of *Nigella sativa *(200 mg/kg, p.o.) for the next eight consecutive days. The animals were fasted overnight after the 7th day treatment. On the morning of, 8th day, the last dose of *Nigella sativa *was administered to the fasted animals. One hour after the last dose of *Nigella sativa, *CsA (30 mg/kg, p.o.) was administered, and the same sampling scheme was repeated as described previously. The blood samples were stored at −80°C until analyzed. 

The same study protocol was followed for *Lepidium sativum *and CsA interaction study, with a difference of *Lepidium sativum* (150 mg/kg p.o.) which was administered orally in place of *Nigella sativa *(200 mg/kg, p.o).

## 3. Bioanalysis of Cyclosporine

### 3.1. Liquid Chromatography and Mass Spectrometric Conditions

The quantitative analysis of cyclosporine A was performed on an acpuity UPLC in tandem with triple quadrupole mass spectrometer (Waters Corp., Milford, MA, USA). The UPLC system included quaternary solvent manager, a binary pump, degasser, and autosampler with an injection loop of 10 *μ*L and a column heater. The drug and internal standard (IS) were eluted on Acquity UPLC BEH C_18_ column (50 × 2.1 mm, i.d., 1.7 *μ*m, Waters, MA, USA) maintained at 38°C. The elution was assisted by mobile phase composed of 20 mM ammonium acetate and methanol (10 : 90), flowing at an isocratic flow rate of 0.25 mL/min. For analysis, 10 *μ*L sample was injected through autosampler. The temperature of autosampler was kept at 20°C.

A triple quadrupole mass spectrometer (Micromass Quattro micro Waters Corp., Milford, MA, USA) equipped with electrospray ionization (ESI) interface was used for quantification of CsA. The ESI source was operated in positive ionization mode. The quantification of CsA was performed using single ion recoding (SIR) mode. Sodium adducts of CsA (m/z 1225.2) and CsD (1239.2) were selected for the quantification. The dwell time of CsA and CsD sodium adducts was 289 ms. Nitrogen was used as a desolvation gas at a flow rate of 600 L/h. The desolvation temperature was 350°C, whereas source temperature was 150°C. The capillary voltage was set at 3 kV. The MS analyzer parameters such as lower mass resolution and higher mass resolution were 10.0 and 14, respectively. The ion energy_1_ and cone voltage were optimized as 1.3 V and 95 V, respectively. The UPLC-MS/MS system was monitored by Mass Lynx software (Version 4.1, SCN 714), and the data was processed using QuanLynx program.

### 3.2. Calibration Standards and Quality Control Samples

Standard stock solutions of cyclosporine A and cyclosporine D (IS) were prepared in methanol to give a final concentration of 1 mg/mL. The solutions were stored in a refrigerator below 8°C and used for 15 days from the date of preparation. Stock solution of cyclosporine A was used to prepare working solutions. Working solutions of cyclosporine A were prepared in methanol : water (50 : 50). The 20 *μ*L aliquot of each working solution was added to 300 *μ*L blank rabbit blood to yield spiked calibration standards ranging from 26.4 to 1705 ng/mL. The quality control (QC) samples at four different concentrations were also prepared in a similar manner as the calibration standards. The IS working solution (5 *μ*g/mL) for routine use was prepared by diluting the cyclosporine D stock solution in methanol-water (50 : 50, v/v). Spiked blood calibration standards and quality control samples were stored at around −80°C until analyzed.

### 3.3. Sample Preparation

The protein precipitation method was used to prepare samples for analysis. Blood samples stored at around −80°C were thawed at room temperature and vortex for 30 sec to ensure homogeneity. To the 300 *μ*L of blood sample, 50 *μ*L (5 *μ*g/mL) of IS (cyclosporine D) was added. The samples were vortex mixed for about 30 sec, and then 150 *μ*L of zinc sulphate (0.2 M) was added to rupture the RBCs, vortex mixed again. The 500 *μ*L of methanol was added to precipitate protein of the lysed blood sample. The samples were again vortex mixed gently for 1.5 min and then centrifuged for 10 min at 12000 rpm. After centrifugation, 400 *μ*L of clear supernatant was transferred into UPLC vials. The 10 *μ*L of each sample was subjected to UPLC-MS analysis.

### 3.4. Pharmacokinetic Analysis

The pharmacokinetic profiles of CsA control and herbs treated groups were plotted between blood concentration of CsA and corresponding sampling time. The pharmacokinetic parameters of control and treated groups were obtained by using PK software on Microsoft Excel. The noncompartmental pharmacokinetic parameters including maximum blood concentration (*C*_max⁡_) and time to reach maximum concentration (*T*_max⁡_), area under the curve from 0 to 24 hrs (AUC_0–24_), from 0 to ∞ (AUC_0–*∞*_), and area under first moment curve from 0–24 (AUMC_0–24_), elimination rate constant (*K*_el_), and half-life were calculated, Whereas mean residence time (MRT) was calculated as the ratio of AUMC_0–24_ and AUC_0–24_. The total body clearance (CL) was estimated by dividing the total administered dose by AUC_0–*∞*_. 

### 3.5. Statistical Analysis

The data is presented as mean with standard error of mean (SEM) for the individual group. Differences in pharmacokinetic parameters of CsA before and after treatment with *Nigella sativa* and *Lepidium sativum *were assessed by a Student's *t*-test. Statistical significance was assumed when *P* ≤ 0.05. 

## 4. Results and Discussion

The *in vivo* investigations were carried out to determine the effect of *Nigella sativa* and *Lepidium sativum* on CsA pharmacokinetics. The pharmacokinetic (PK) profile of CsA was expressed by plotting its blood concentration versus time curve. The PK parameters of CsA control group were compared with *Nigella sativa* and *Lepidium sativum* treated groups. 

**Figure 1:**
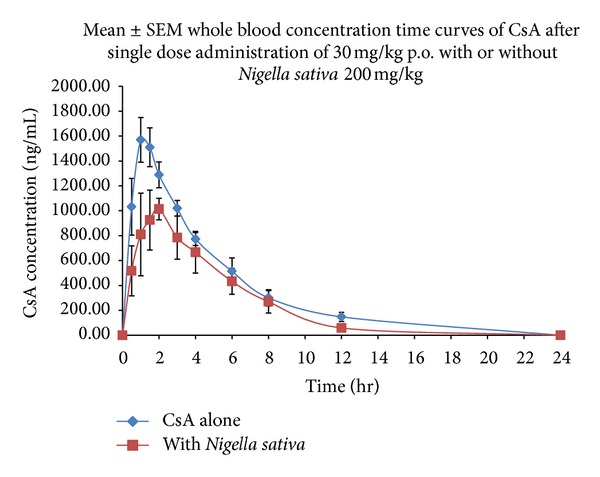
The blood concentration versus time profiles of cyclosporine A control and *Nigella sativa* treated group.

The mean blood concentration versus time curves of CsA (30.0 mg/kg, single oral dose) control and *Nigella sativa* (200 mg/kg p.o.) treated group is illustrated in Figure 1. The corresponding pharmacokinetic parameters of CsA control and *Nigella sativa* treated group are summarized in Table 1. The observations showed that mean maximum blood concentration and area under curve of CsA was significantly decreased when coadministered with *Nigella sativa*. The other PK parameters such as *T*_max⁡_, Vd and MRT did not show remarkable influence of *Nigella sativa. *In *Nigella sativa *treated group, the *C*_max⁡_ and AUC_0–*∞*_ of CsA were decreased by 35.5% and 55.9%, respectively (*P* ≤ 0.05). The *T*_max⁡_ was delayed from 1.20 h to 1.88 h, but the difference was not statistically significant. Insignificant difference was observed in the *T*_1/2_ (3.28 to 2.96) and MRT (4.69 to 4.62) of CsA control and *Nigella sativa* coadministered group, respectively. A statistically significant (*P* ≤ 0.05) increase in CsA CL (3.32 to 6.27 L/kg) was observed after *Nigella sativa* treatment, however the increase in Vd was statistically insignificant (15.45 to 28.25 L/kg). 

**Figure 2:**
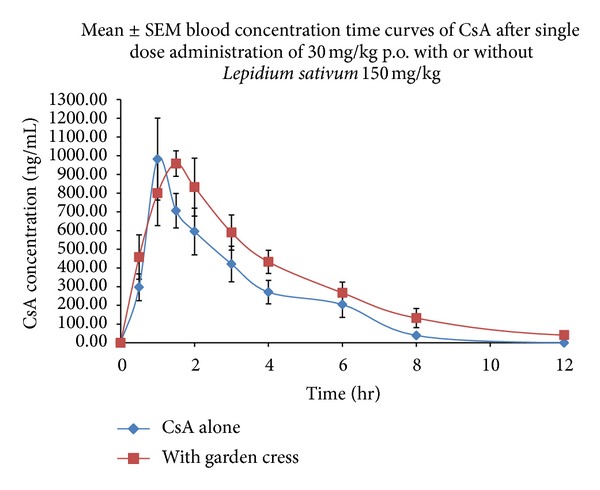
The blood concentration versus time profiles of cyclosporine A control and *Lepidium sativum* treated group.

The mean blood concentration versus time curves of CsA (30.0 mg/kg, single oral dose) control and *Lepidium sativum* (150 mg/kg p.o.) treated group is illustrated in Figure 2. The corresponding pharmacokinetic parameters of CsA control and *Lepidium sativum* group are summarized in Table 2. The coadministration of *Lepidium sativum* did not produce any significant impact on the maximum blood concentration of CsA. The *C*_max⁡_ of CsA control (999.76 ± 260.54) and *Lepidium sativum* treated group (984.04 ± 79.42) was found to be almost equal. The data obtained from *Lepidium sativum* treated group showed that time to reach maximum blood concentration was significantly higher (1.75 hours) than control group (1.13), (*P* ≤ 0.01). It is clear from Figure 2 that concurrent administration of *Lepidium sativum* increased the AUC_0−*t*_ and AUC_0–*∞*_ of CsA by about 40%, but the change was not statistically significant. Taking into consideration the information derived from PK profile, the half-life of CsA was increase in presence of *Lepidium sativum.* The half-lives of CsA in *Lepidium sativum* treated and control groups were found to be 1.96 and 1.59 hr (*P* ≤ 0.05), respectively.  *Lepidium sativum* decreases the elimination rate of CsA from 0.44 to 0.37 hr^1^ (*P* ≤ 0.05). *Lepidium sativum* did not show a statistically significant effect on CL (12.13 to 8.67 L/kg) and Vd (27.86 to 23.87 L/kg). 

Several interaction studies of CsA with herbal products have been described in different animal models. Coadministration of herbs has been reported to modulate the oral bioavailability of CsA [14–16, 18, 26, 27]. To the best of our knowledge, this is the first study designed to evaluate the effect of *Nigella sativa *and *Lepidium sativum* on the pharmacokinetics of orally administered CsA in rabbits. Our results showed that bioavailability (both rate and extent) of CsA in rabbits decreased significantly after oral treatment with *Nigella sativa*. The elimination rate and half-lives of CsA alone and coadministration with *Nigella sativa* were not significantly different. Thus, it can be suggested that the decreased bioavailability of CsA caused by *Nigella sativa* may occur at absorption site, apparently due to the significant increase in CsA clearance (~2-fold). The modulation of mechanisms affecting the fate of CsA at absorption site (P-gp and CYP3A4 in intestine) may be the reason of reduced absorption. Therefore, reduced oral absorption of CsA in the presence of *Nigella sativa* may be associated with the activation of intestinal P-gp and/or CYP3A4.

Coadministration of *Lepidium sativum* showed moderate effect on the disposition profile of CsA in rabbits. Without showing any remarkable effect on *C*_max⁡_, a significant prolongation of *T*_max⁡_ was observed in *Lepidium sativum* treated group. Although the AUC_0–24_ and AUC_0–*∞*_ of *Lepidium sativum* treated group were increased more than 40%, this was not statistically significant, which may be due to the high variability in data. The coadministration of *Lepidium sativum* reduces the CsA clearance (~1.4-fold), but the change was not significant. The elimination rate of CsA coadministered with *Lepidium sativum* was significantly decreased, though the half-life and *T*_max⁡_ were increased. The decreased elimination rate suggested the inhibition of CYP3A and modulation of P-gp in presence of *Lepidium sativum.* Further, the increased oral bioavailability of CsA in presence of *Lepidium sativum* can be associated with CYP3A and P-gp.

The interspecies and interindividual variation in cyclosporine absorption and metabolism among rabbits, rats, human, and dogs were in agreement with a previous report and could be explained by several interrelated factors and interindividual variability of cytochrome-P450 and P-gp activities [28–32]. The present studies have used crossover designs to conquer this problem, but still high variability was observed. Although the AUC_0–*t*_/AUC_0–*∞*_ (%) ratios for both studies were more than 80%, emphasizing the duration of sample collection was sufficient to characterize the pharmacokinetic profiles. 

## 5. Conclusion

Our findings suggest that *Nigella sativa *can significantly alter CsA pharmacokinetics *in vivo* possibly by modulating the activities of CYP3A and P-gp. *Lepidium sativum *did not greatly affect the disposition profile of CsA in rabbits. The markedly decreased *C*_max⁡_ and AUC_0–*t*_ of cyclosporine caused by coadministration with *Nigella sativa* indicated that the oral bioavailability of CsA was significantly reduced, whereas *Lepidium sativum* improved the AUC_0–*t*_ of CsA, showing increased bioavailability. Based on these results, precaution should be taken in mind to avoid coadministration of these herbs with CsA until a lack of significant interaction in human is proven by appropriately designed clinical studies.

## Figures and Tables

**Table 1:** Pharmacokinetic parameters of CsA control (alone) and coadministered with *Nigella sativa* (mean ± SEM, *n* = 5).

Parameters	CsA alone (mean ± SEM)	With *Nigella sativa* (mean ± SEM)
^ a^ *C*_ max_ (ng/mL)	1597.15 ± 168.54	1177.94 ± 168.24*
^ b^ *T*_ max_ (hr)	1.20 ± 0.11	1.88 ± 0.43
^ c^AUC_0–24_ (ng·hr/mL)	8378.37 ± 430.92	5809.81 ± 1217.46*
^ d^AUC_0–*∞*_ (ng·hr/mL)	9202.32 ± 522.10	5893.04 ± 1267.95*
^ e^Kel (hr^−1^)	0.24 ± 0.04	0.24 ± 0.02
^ f^ *T*_1/2_ (hr)	3.28 ± 0.48	2.96 ± 0.22
^ g^CL (L/Kg)	3.32 ± 0.20	6.27 ± 1.92*
^ h^MRT (hr)	4.69 ± 0.37	4.62 ± 0.36

*Significant difference from “CsA alone” group with *t*-test,*P ≤ 0.05, ^a^maximum blood concentration, ^b^time of peak concentration, ^c^area under the concentration time profile curve until last observation, ^d^area under the concentration time profile curve from time 0 to infinity, ^e^elimination rate constant, ^f^half-life, ^g^Totalclearance, and ^h^mean residence time (MRT).

**Table 2:** Pharmacokinetic parameters of CsA control (alone) and coadministered with *Lepidium sativum* (mean ± SEM, *n* = 5).

Parameters	CsA alone (mean ± SEM)	With garden cress (mean ± SEM)
^ a^ *C*_ max_ (ng/mL)	999.76 ± 260.54	984.04 ± 79.42
^ b^ *T*_ max_ (hr)	1.13 ± 0.11	1.75 ± 0.13**
^ c^AUC_0–24_ (ng·hr/mL)	2790.81 ± 541.71	3965.47 ± 609.20
^ d^AUC_0–*∞*_ (ng·hr/mL)	2795.67 ± 542.28	3974.74 ± 606.11
^ e^Kel (hr^−1^)	0.44 ± 0.02	0.37 ± 0.03*
^ f^ *T*_1/2_ (hr)	1.59 ± 0.08	1.96 ± 0.18*
^ g^CL (L/Kg)	12.13 ± 2.04	8.67 ± 1.98
^ h^MRT (hr)	2.92 ± 0.13	3.49 ± 0.39

*Significant difference from CsA alone group with *t*-test, *P ≤ 0.05, **P ≤ 0.01. ^a^maximum blood concentration, ^b^time of peak concentration, ^c^area under the concentration time profile curve until last observation, ^d^area under the concentration time profile curve from time 0 to infinity, ^e^elimination rate constant, ^f^half-life, ^g^totalclearance, and ^h^mean residence time (MRT).
